# Exploring the Cultural Adaptation of an Ongoing Evidence-Based Intervention for Chinese and Korean American Dementia Caregivers: Descriptive Study

**DOI:** 10.2196/86499

**Published:** 2026-04-23

**Authors:** Eunjung Ko, Bei Wu, Jing Wang, Xiang Qi, I Tek Leong, Yaolin Pei, Weiyu Mao, Jin Su, Feitong Zhang, Lindawest Wang, Cynthia Epstein, Mary S Mittelman

**Affiliations:** 1 Rory Meyers College of Nursing, New York University, 433 1st Avenue, New York, NY, 10010, United States, 1 212-998-2398; 2 Art and Sciences, New York University Shanghai, Shanghai, China; 3 College of Health and Human Services, University of New Hampshire, Durham, NH, United States; 4 Silver School of Social Work, New York University, New York, NY, United States; 5 School of Nursing, The University of Texas at Austin, Austin, TX, United States; 6 School of Social Work, University of Nevada, Reno, Reno, NV, United States; 7 Graduate School of Education, Harvard University, Cambridge, MA, United States; 8 Grossman School of Medicine, New York University, New York, NY, United States

**Keywords:** dementia, caregivers, evidence-based intervention, program descriptions, Asian, culture

## Abstract

**Background:**

The aging and caregiving population is becoming increasingly diverse in the United States, leading to a growing need for culturally adapted interventions to address the unique needs of underrepresented groups, such as Asian Americans. However, interventions targeting Asian Americans and exploring cultural adaptation strategies remain limited in dementia caregiving research.

**Objective:**

This study aimed to describe the cultural adaptation process of an evidence-based intervention for Chinese and Korean American dementia caregivers, called the New York University Caregiver Intervention–Enhanced Support.

**Methods:**

We conducted a deductive content analysis and categorized our adaptation strategies into 5 elements: content, context, relationship fidelity and core elements, engagement, and cultural competence. Timing and types of responses to each adaptation strategy were also observed. Two authors conducted the initial analysis, and additional team members finalized the synthesis through discussion. The Template for Intervention Description and Replication (TIDieR) checklist was used to guide the methodological rigor.

**Results:**

Twenty-four major adaptations were identified and categorized. For content, we translated materials, used culturally relevant terms, incorporated ethnic-specific surveys and resources, created social media support groups on platforms widely used by the targeted population, and extended the time allocated to complete the 6 counseling sessions. Context adaptation included expanding the range of individuals eligible for family counseling sessions to include fictive kin, using online and social media apps for communication, cultural matching and training of staff, and partnerships with relevant community organizations. Relationship fidelity and core elements involved consulting with community experts, conducting focus group interviews with caregivers, having regular meetings with the developer of the original intervention and an experienced New York University Caregiver Intervention–Enhanced Support clinician as well as experts in Chinese and Korean culture, and continuing regular counseling supervision. To enhance engagement, we provided clear explanations of the study procedure, which emphasized the benefits in participants’ native languages and matched participants with social workers who shared the same cultural backgrounds. We also used a step-by-step contact approach and prolonged communication, explained staff roles to build rapport, and offered participant compensation. Finally, cultural competence was reflected in tailoring counseling techniques with respect for cultural beliefs, the use of euphemistic language for taboo subjects, and culturally appropriate refreshments to show respect and build interpersonal relationships.

**Conclusions:**

We systematically adjusted a counseling-based intervention, an approach less familiar among Asian Americans, to fit the cultural characteristics of the target population. A contribution of this study is using an integrated, theory-driven approach that combines 2 cultural adaptation frameworks while also capturing real-time adaptations informed by external feedback and self-reflection. This work provides a practical model for adapting evidence-based interventions to serve Chinese and Korean American dementia caregivers and may inform future adaptations for other East Asian populations.

## Introduction

### Background

As the US caregiver population becomes increasingly diverse, including an increasing number of Asian Americans, there is a critical need to develop and implement culturally tailored interventions that address the unique values, beliefs, and needs of underrepresented groups [1,2]. The importance of cultural adaptation is underscored by findings that interventions may not be equally effective across all populations due to cultural differences [3,4]. Cultural adaptation can enhance intervention effectiveness for different groups by making them culturally relevant, reducing health disparities, and offering more comprehensive support [1,4,5]. Interventions targeting the needs of different races and cultures have recently been developed and monitored; however, Asian Americans are the least targeted group for interventions [6,7], including interventions for dementia caregivers from Asian cultures [8-10]. Culturally tailored interventions for dementia caregivers from Asian backgrounds living in the United States warrant further modification in terms of implementation guidelines, evaluation, and intervention provider training [7].

In response to this gap, we adapted the New York University Caregiver Intervention (NYUCI) for Chinese and Korean American dementia caregivers, named NYUCI-Enhanced Support (NYUCI-ES) [10,11]. The original NYUCI was developed in the 1980s to improve caregiver well-being and delay nursing home placement for individuals living with dementia [12-14]. The intervention was originally evaluated in a randomized controlled trial (RCT) and targeted spousal or partner caregivers of individuals with dementia residing in the New York City metropolitan area. Participants in the treatment group received 2 individual and 4 family counseling sessions in 4 months after baseline and were encouraged to attend community support groups and use ad hoc (on-request) counseling from their assigned counselor. The content and duration of counseling sessions varied according to participants’ interests and availability. In contrast, those in the control group received ad hoc counseling and resource information based on their needs but did not participate in the 2 individual and 4 family counseling sessions provided to the treatment group; participants were evaluated every 4 months for the first year and every 6 months thereafter [12,13].

The intervention helped reduce depressive symptoms and stress among caregivers in the treatment group and improved their subjective assessment of physical health [12,15]. It also delayed nursing home placement for individuals with dementia whose caregivers were in the treatment group by a median of 557 days [13]. The intervention was later applied to the population of adult children caring for parents living with dementia and revealed similar positive outcomes [16,17]. These results contributed to its implementation across the United States and elsewhere [15,17-21]. NYUCI was also adapted to Hispanic caregivers in New York City, whose family support has a significant role in caregiving [22], similar to the Asian population [10]. The intervention has demonstrated significant reductions in caregiver burden in the Hispanic population [23].

Given the potential of the NYUCI to support a diverse population and the current lack of culturally tailored support for Asian American communities [10,11], it is important to discuss how we conducted systematic, real-time cultural adaptations made to the NYUCI model to align with Chinese and Korean cultural values and support the feasibility of the intervention in the target population (ie, NYUCI-ES). In this study, we first describe Eastern cultural perspectives on caregiving, as well as previously developed culturally adapted interventions and adaptation frameworks. We then document the cultural adaptation strategies applied to the original NYUCI procedures before and during implementation using these established frameworks. Our focus herein is on the adaptation process; participant outcomes will be reported separately at a later time following study completion.

### Eastern Cultural Perspectives on Caregiving

Asian Americans are the fastest-growing ethnic group in the United States, expected to reach 46 million by 2060 [24]. Compared to other racial groups, Asian American dementia caregivers are more likely to find the consideration of cultural background in care settings important but perceive a lack of a culturally competent health care system, along with challenges in care navigation [25]. As the majority of Asian American older adults were born outside the United States [24], this racial group also tends to experience acculturative stress and family conflicts as they maintain traditional culture while assimilating into a new society [26].

Individuals originating from East Asian countries, including Chinese and Korean, account for 37% of the Asian American population [27]. Although Asian Americans exhibit high cultural heterogeneity among subgroups in terms of language, religion, and historical backgrounds [27], those from East Asian countries tend to share more similar cultural traits due to geographical proximity and historical closeness [28]. Specifically, their cultural similarities result from Confucianism, which emphasizes benevolence, integrity, propriety, morality, and trust [29]. This can also lead to cultivating oneself to be morally perfect and become part of society, prioritizing family values over personal ones, controlling oneself in behaviors tied to filial piety, and expressing respect and loyalty toward older adults [29,30].

These values often lead to assigning caregiver roles to family members, particularly children of the family member living with dementia [31], who feel a greater sense of filial obligation to reciprocate the previous care that they received [26]. Familism also requires individuals to follow family decisions rather than their own decision-making process, resulting in a disregard for individuals’ emotions and needs [28,31]. In addition, dementia caregivers from East Asian cultures are likely to have the misconceptions that dementia symptoms are a normal part of aging or a matter of fate [32,33]. Stigma against dementia is often confounded with stigma against mental illness [26,34,35]. These perceptions can delay treatment for dementia [33], causing emotional stress, burden, and negative health status among family caregivers [36].

### Existing Culturally Adapted Interventions Incorporating East Asian Culture

A few previous researchers have adapted interventions into Eastern cultural contexts to take into account differences from Western cultural norms [9,37-41]. For instance, Jang et al [9,41] adapted the Savvy Caregiver Program for Korean American dementia caregivers (K-Savvy) using the cultural adaptation process of Barrera et al [5] and the Framework for Reporting Adaptations and Modifications–Enhanced (FRAME) [42]. They translated study materials into Korean, reframed program content to align with collectivist cultural values, extended the length of sessions based on participants’ needs, collaborated with multiple stakeholders, and trained staff regarding the program components and an appropriate culturally related approach [9,41]. Similar strategies were used in other adapted interventions, along with modifications to the delivery format, such as omitting phone-based sessions [37], implementing an in-home approach [39], and using a DVD delivery format [38,40].

While prior studies have incorporated cultural elements into dementia caregiver interventions, few have used a structured framework to assess adaptation strategies, which is essential for understanding the mechanisms and evaluation of the strategies [43]. In addition, most research addressed preimplementation modifications rather than exploring ongoing adaptations made during the implementation based on participants’ feedback and discussions with the research team. This limits the ability to refine interventions during implementation.

### Cultural Adaptation Framework for Intervention Implementation

Stirman et al [42] developed FRAME, which provides a systematic structure to assess how and why interventions are modified, improving the transparency as well as effectiveness of the adaptation. It represents an expanded version of their previous framework and incorporates pivotal components to consider during the adaptation process. These components contain intervention elements being modified, the individuals who are involved in the modification, the timing (ie, preimplementation, implementation, scale-up, and sustainment), and types of responses (ie, proactive vs reactive) to the modification, the changes in delivery methods, and the underlying reasons for modification—including cultural norms.

To narrow down the intervention type and focus more on the adaptation of intervention elements, Day et al incorporated FRAME into a systematic review of cultural adaptation frameworks for psychosocial interventions and addressed the literature gap regarding limited details on the components for cultural adaptation. They synthesized the findings using FRAME as part of their analytic structure and identified 5 core elements: *content*, *context*, *relationship fidelity and core elements*, *engagement*, *and cultural competence*. “Content” involves changes in the intervention materials and procedures, including adding or modifying original program components, changes to or translations of study materials, and adjustments in the number or intervals of the interventions. “Context” refers to modifications in the intervention’s setting or personnel while preserving its core “content,” such as adjusting delivery methods, providing staff training, and considering community and situational factors. “Relationship fidelity and core elements” considers how interventions lead to different intervention outcomes due to participant diversity. It emphasizes the importance of adapting the intervention to align with the target population’s cultural values while preserving its core elements to maintain the effectiveness of the original intervention. “Engagement” reflects the process of building participants’ relationships with researchers and the intervention. It encompasses strategies to effectively recruit and retain participants by enhancing awareness, accessibility, and trust in both the intervention and its providers. Finally, “cultural competence” describes the ability to integrate cultural norms and attitudes to implement interventions effectively in the targeted cultural settings. It also includes the capacity to provide individualized accommodations throughout the intervention [2].

The framework of Day et al [2] underscores the importance of maintaining intervention fidelity and promoting cultural relevance while adapting evidence-based psychosocial interventions across diverse populations, offering a basis for evaluating culturally adapted interventions. However, the synthesis was specific to intervention elements and did not consider the time, which could affect whether certain adaptations were feasible for participants during the implication phase. Considering time-related factors in addition to Day’s 5 elements would give researchers a more concrete understanding of cultural adaptation strategies.

### Study Aims

Given the gaps in knowledge and a pressing need for cultural adaptation in interventions, this study explored the process of culturally adapting an enhanced version of the original NYUCI for 2 East Asian subgroups. To be specific, this study identified the types of cultural adaptations made to the intervention for each of the targeted subgroups and described when these adaptations occurred throughout the implementation process.

## Methods

This study used a descriptive research design and used deductive content analysis. To guide the cultural adaptation process, this study drew on both the framework of Day et al [2] and the FRAME [42], as each framework addresses distinct but complementary aspects of cultural adaptation.

### Parent Study: NYUCI-ES

The NYUCI-ES, an ongoing study of an evidence-based intervention using an RCT design, aimed to assess its effects on improving the physical and psychological health of Korean and Chinese American caregivers who have risk factors for cardiometabolic diseases. To be eligible, participants should be primary caregivers of a family member living with memory problems in community settings and aged 50 years or older. They should also have Chinese or Korean ancestry, live in the New York City metropolitan area, have access to the internet as well as a phone with voice and text messaging capabilities, report that they have at least one family member who can attend family counseling sessions, have sufficient proficiency in English, Chinese, or Korean, and have at least one of the self-reported medical conditions related to cardiometabolic syndrome, such as diabetes, high cholesterol, high blood pressure, obesity, and/or other related symptoms. The target was 150 Chinese American and 150 Korean American participants, with a sample size estimated to provide 80% power to detect small to moderate effect sizes (ranging from 0.22 to 0.31) in key inflammation biomarkers using an analysis of covariance model, consistent with benchmarks defined by Sawilowsky [44]. Recruitment was conducted through, but was not limited to, community organizations, including hospitals, clinics, and adult day care centers; word of mouth, community outreach and events, and media advertisements; and a research registry targeting Asian Americans, Native Hawaiians, and Pacific Islanders [45,46]. Participant recruitment began in February 2023 and was completed by September 2025.

After being deemed eligible and completing informed consent and the baseline assessment visit, participants were randomized in a 1:1 ratio to either the treatment or control group using a computer-generated allocation sequence. Participants in the treatment group received (1) a total of 6 counseling sessions—2 individual (between the participant and a social worker) and 4 family counseling sessions (among the participant, at least one family member, and a social worker)—in person, via video calls, or by phone, with flexible session lengths depending on participants’ needs; (2) ad hoc counseling that is initiated at the participant’s request; and (3) enhanced support through an online chat group (eg, WeChat for Chinese participants and KakaoTalk for Korean participants), which is a newly added feature to the original NYUCI. Participants completed the counseling sessions before the second visit but can continue to use ad hoc counseling sessions and join the online chat group throughout the 1-year study period. They were also recommended to join community support groups, as in the original NYUCI, to access additional supportive resources. Caregivers in the control group are not offered structured counseling sessions but may use ad hoc counseling for caregiving resources. They can also participate in an online study chat group for enhanced support throughout the study. We assessed participants’ physical and psychosocial status at baseline, 6 months, and 12 months using written assessments and biometric tests, including measures of chronic disease management, stress, depression, blood pressure, BMI, and metabolic biomarkers. Detailed study information is provided in Table 1 and elsewhere [11].

**Table 1.** Study procedure of New York University Caregiver Intervention–Enhanced Support (NYUCI-ES)

Characteristics	NYUCI-ES
Study period and design	A year-long randomized controlled trial (ongoing)
Study purpose	To assess the effects of NYUCI-ES in improving the physical and psychological health of Korean and Chinese American caregivers who have risk factors for cardiometabolic diseases
Study population (eligibility)	Korean or Chinese Americans aged 50 years or older who live in the New York metropolitan area Those who provide care for people living with memory problems in the community setting Those who can access the Internet as well as phone with voice messages and text messages Those who report that they have at least one family member who can attend family counseling sessions Those who have sufficient proficiency in English, Chinese, or Korean Those who have at least one cardiovascular disease risk factor (eg, obesity, diabetes, hypertension, hyperlipidemia, and other related symptoms)
Recruitment strategies	Community organizations, word of mouth, community outreach and events, media advertisement, a research registry, and so on
Study procedure	Treatment and control groups: Three assessment visits (baseline, 6-mo follow-up, and 12-mo follow-up) Caregiver-initiated counseling as needed (ie, ad hoc counseling) Participation in a social media chat group Treatment group only: Six counseling sessions (2 individual and 4 family-based counseling sessions) (between first and second visits)
Compensation	US $150 to US $200 (US $50 per assessment visit and US $50 for completing the metabolic biomarker test during each assessment visit)

### Data Collection

For the cultural adaptation, adaptations before starting the intervention phase of the study had been proposed through team discussions, focus group interviews of eligible individuals [10], and input from the community advisory board. Ongoing adaptation was a result of information from consultation sessions with community partners, weekly team meetings, meeting notes, self-reports from the staff who communicate with participants or the senior counselor, and team members’ observations during follow-up interview visits and counseling sessions. The information gathered by the aforementioned sources was reviewed and summarized to identify topics and to match the categories of Day et al [2]. The community advisory board, composed of community organization staff, clinicians, and professors, provided ongoing feedback on cultural relevance throughout the preimplementation period. The modifications during the implementation period were primarily based on staff observations, experiences encountered during trial delivery, and active discussions with all study team members, including the original program developer and a senior counselor.

### Data Analysis

The coding and categorization procedures described below were used to document and analyze the cultural adaptation process conducted as part of the NYUCI-ES. We used deductive content analysis to operationalize the concept of previous knowledge [47]. This analysis process began with the development of an analysis matrix, followed by data collection and categorization guided by the analysis matrix [47]. On the basis of the 5 elements of Day et al [2] and the FRAME’s timing and type of response to adaptation [42], we analyzed our cultural adaptation approach. The adaptation strategies we implemented were first summarized by 2 research team members (EK and AL) who shared the same cultural backgrounds as the participants and served as frontline staff, using Microsoft Word and Excel. After gathering the strategies, they were reassessed and categorized based on the 5 elements of Day et al [2]. The coding was independently conducted. The 2 research team members participated in iterative discussions to resolve discrepancies and refine the categorization.

Following this content-based categorization of the cultural adaptation strategies, the FRAME was applied to further characterize each adaptation in terms of its implementation process. For instance, each adaptation strategy was identified by when it occurred along the implementation timeline (eg, preparation vs implementation) and whether it was planned (eg, proactive vs reactive). This analytic approach systematically identified culturally grounded adaptation strategies and characterized them using established frameworks, thereby directly addressing the aim of the study.

The other 2 team members (YP and XQ), who have a Chinese cultural background and had previously served as program managers for this study, reviewed the coding decisions and categorizations, resolved any unresolved disagreements, and provided suggestions if necessary. The Template for Intervention Description and Replication (TIDieR) checklist was used to ensure methodological rigor [48].

### Ethical Considerations

The parent study was approved by the Institutional Review Board of New York University (i22-00615) and registered on ClinicalTrials.gov [49] on July 18, 2022. Participants in the NYUCI-ES provided informed consent before the data collection. They were informed about the study procedure, the deidentification of their data during analysis to protect privacy and confidentiality, limited access to their data within the research team, the potential benefits and risks of participation, and the contact information for the principal investigators. Participants received US $50 per visit (US $150 for 3 visits) and an additional US $50 for measuring metabolic biomarkers.

## Results

We found 24 major adaptation strategies and categorized them into 5 key cultural adaptation elements as defined by Day et al [2]. We also identified the timing and types of responses to adaptation by the FRAME framework [42]. The explanation is illustrated in Table 2.

**Table 2.** Classification of the cultural adaptation process based on key adaptation elements (Day et al) [2] and the timing and types of response to adaptations using the FRAME^a^ (Stirman et al) [42].

Adaptation elements [2]	Types of adaptation [2]	Our approach	Timing of response [42]	Type of response [42]
Content: changing materials or procedures of interventions	Changes in packaging or materials Tailoring, tweaking, refining Adding or removing elements Pacing or timing	Translating materials and providing multilingual support Using culturally appropriate terminology, images, questionnaires, and community resources Adding familism, stigma, and social support in surveys Providing social media support groups using popular apps among the Chinese and Korean Extension of the interval of each family counseling session (6 family sessions for 4-6 mo)	Preparation and implementation Preparation and implementation Preparation Preparation Preparation	Proactive Proactive and reactive Proactive Proactive Proactive
Context: modifying the format, setting, personnel, and target population in the intervention	Format Setting Personnel Population	Allowing various fictive kin to join the family counseling Contacting participants in person and online, including through popular social media apps among Chinese or Korean Arrangement of the staff with the same cultural background Attending training sessions about caregiving in Korean or Chinese population Partnering with culturally tailored organizations	Implementation Preparation and implementation Preparation and implementation Preparation and implementation Preparation and implementation	Reactive Proactive and reactive Proactive Proactive Proactive
Relationship fidelity or core elements: ensuring fidelity while preserving key active components during adaptations	—^b^	Having focus group interviews with caregivers who may be eligible for NYUCI-ES^c^ Consulting external community board members with expertise in cultural adaptation Having weekly meetings with the original intervention developer and research personnel with expertise in Korean or Chinese culture Weekly clinical supervision by a senior counselor who was involved in the original intervention	Preimplementation Preimplementation and implementation Preparation and implementation Preparation and implementation	Proactive Proactive Proactive and reactive Proactive and reactive
Engagement: facilitate participants to be involved and remain in the study	Awareness Access or entry Client or therapist relationship Retention or completion	Providing detailed study information in participants’ preferred language and emphasizing the benefits of the intervention Offering clear explanations for the randomization assignment to help participants better understand the study procedure and reduce hesitancy Using a step-by-step outreach process Prolonged, multiple communications in their preferred language Improving credibility through the explanation of staff roles and building rapport through active engagement Offering participant compensation	Implementation Implementation Preparation and implementation Preparation and implementation Preparation and implementation Preparation	Proactive Reactive Proactive and reactive Proactive Proactive and reactive Proactive
Cultural competence: encompassing cultural characteristics effectively in the intervention	—^b^	Delivering culturally, individually tailored counseling Respecting their cultural beliefs Using euphemistic language during communication Providing culturally appealing refreshments during visits	Preparation and implementation Preparation and implementation Preparation and implementation Implementation	Proactive and reactive Proactive and reactive Proactive and reactive Proactive and reactive

a FRAME: Framework for Reporting Adaptations and Modifications–Enhanced.

b Not applicable.

c NYUCI-ES: New York University Caregiver Intervention–Enhanced Support

### Content

While the original NYUCI had flexibility based on participants’ needs, the most important consideration regarding the content was addressing language barriers. Before implementing the study, we translated all materials, such as consent forms, flyers, and questionnaires, from English into simplified Chinese and Korean. During implementation, our team members who were fluent in Chinese and able to read and write both simplified and traditional Chinese added a traditional Chinese version of the study materials to attract participants who use traditional but not simplified Chinese (ie, individuals from Hong Kong or Taiwan). In addition to language, we also carefully considered cultural perceptions of dementia to reduce stigma while selecting wording. For example, we used softer terms, such as “memory loss” instead of “dementia” and “consulting,” “meeting,” “support,” and “education” instead of “counseling.” This linguistic adaptation aimed to make the intervention more culturally acceptable and less intimidating for participants. This was also helpful to our recruitment efforts, addressed elsewhere in relation to this intervention [11]. Additionally, culturally specific questionnaires regarding familism, affiliated stigma against dementia, and acculturation status were included to embrace caregivers’ traditional culture during the preparation period.

To enhance both formal and informal support, we integrated regular online support group sessions into the original NYUCI program using social media apps (eg, WeChat for many Chinese participants and KakaoTalk for the Korean participants) that are more commonly used apps among the target population. Through this platform, we delivered weekly educational resources on dementia care, self-care strategies, and available community resources where participants could seek help from culturally competent organizations. We also facilitated peer connections through the group chats, allowing participants to share experiences and support one another. Regarding the pacing and timing, family counseling sessions were modified by extending the phase of 6 counseling sessions in the original NYUCI, from 4 to 6 months. This strategy was adapted from the earlier adaptation of NYUCI targeting the Hispanic population [22], and NYUCI-ES applied a similar adaptation approach to accommodate participants’ availability.

### Context

For family counseling sessions, we used a broad definition of family. In response to implementation-related challenges involving participants who had family members eligible to join but preferred not to include them in the family sessions, we allowed participants to invite a range of individuals who had close bonds with them (ie, fictive kin). This range included family members or relatives living in other states or overseas, friends, neighbors, and local community organization staff participants whom they frequently relied on. These individuals shared similar cultural or situational backgrounds with the participants, which made their involvement feel natural and supportive.

The NYUCI was adapted for telehealth in Virginia and, in a pre-post study, demonstrated favorable outcomes, including reductions in caregivers’ burden, depressive mood, and reactions to their care recipient’s symptoms [20]. Similar to this adaptation, we offered counseling sessions either in person or online, as preferred, to accommodate participants’ schedules and locations and to reduce the burden of attending in person. During implementation, we also used the aforementioned culturally familiar social media apps, such as WeChat or KakaoTalk, to communicate with participants more conveniently.

Additionally, we matched frontline team members who shared the same cultural and linguistic backgrounds as the participants. Before and during implementation, bilingual social workers and research assistants who were fluent in Chinese (Mandarin and Cantonese) or Korean and had in-depth knowledge about their respective cultures were hired. Social workers also received training in caregiving approaches specific to Chinese and Korean populations, thereby enhancing the effectiveness of culturally tailored counseling. Moreover, we collaborated with and maintained partnerships with community organizations that serve these populations in New York City. This partnership was sustained through occasional meetings with community partners or community advisory members, participation in community events, and the provision of educational presentations.

### Relationship Fidelity and Core Elements

While integrating Korean and Chinese cultural values into the original NYUCI program, we made efforts to maintain the core concepts. First, during the preparation period, we conducted a focus group interview with 25 caregivers of people living with dementia (14 Chinese and 11 Korean) to explore their caregiving experiences and needs that could inform the adaptation of the intervention [10]. We also received consultation from community experts, including faculty members in academic institutions, clinicians, and community partners, through occasional meetings to improve recruitment and retention and to discuss adaptation strategies.

We also held weekly meetings with key team members, including the original intervention developer, lead counselor, research staff with expertise in Chinese or Korean culture, and social workers who mainly interacted with the participants. These meetings have been continued during the preimplementation and implementation processes. During the implementation period, frontline team members shared updates on recruitment, implementation, counseling sessions, and any challenges faced while communicating with participants. All team members discussed possible solutions during meetings or through email between meetings. The original program developer and senior counselor revisited examples that reflected local family structures based on their previous applications of the intervention, compared them with this study, and confirmed that adjustment of the content of sessions to reflect the cultural context was within the spirit of the original intervention’s purpose. In addition, social workers responsible for counseling also received additional weekly clinical supervision sessions from a senior counselor with expertise in the original intervention to discuss participants’ concerns or needs during the intervention and provide culturally aligned counseling while preserving the values and contents of the original interventions.

### Engagement

We offered detailed explanations of the study’s purposes, procedures, and expected outcomes in participants’ preferred language to build trust and ensure participants’ understanding and adherence to the NYUCI-ES. For example, to align with the core East Asian value of family harmony, we emphasized that the primary goal of counseling was to maintain and strengthen family harmony as well as the caregivers’ own capacity. We also highlighted the potential benefits of counseling sessions, which are often unfamiliar in Chinese and Korean cultures, and personalized the topics that would be addressed in the counseling sessions. This effort was reiterated throughout the screening calls, baseline interviews, and initial individual and family counseling sessions by frontline team members. We also provided detailed information about the importance of RCTs. Participants tended to have limited familiarity with research and hesitated to join the study in case they would be assigned to the treatment group, potentially leading to scheduling conflicts and perceived burden. During the initial orientation session, we emphasized that assignment to the treatment or control groups would occur by chance (eg, “through the computer”) rather than by participant choice or staff preference, and that this procedure is necessary to assess how effective the intervention would be for their health and to further provide evidence-based support for other Chinese and Korean dementia caregivers. Although a few participants initially expressed concerns about the RCT procedure, these concerns were addressed through repeated clarification of the rationale for the random assignment process.

In addition, we used a step-by-step communication approach to reduce apprehension and encouraged engagement, initially contacting them through text messages or email to introduce ourselves and the study. To enhance the relationship between research staff and participants, we maintained multiple, prolonged communications during screening calls, interviews, and counseling sessions. We also emphasized transparency by clearly stating our job titles and the roles of the intervention team to help participants understand how each team member could support them. Furthermore, we provided support and education for all participants by actively offering them support and engagement through social media platforms to maximize their access to the resources that we could provide and to make participants feel cared for and supported, strengthening the rapport between the study team, specifically, frontline team members. Finally, we provided compensation in the form of physical gift cards, offering US $50 per data collection at baseline and at 6-month and 12-month follow-ups. This compensation was to acknowledge caregivers’ time and efforts, encouraging their consistent engagement and retention in this study.

### Cultural Competence

To appropriately respect and adapt the intervention to Chinese and Korean culture, social workers provided a culturally tailored approach during counseling. In family counseling sessions, we adapted our approach by prioritizing practical caregiving skills, equipping caregivers with the tools they need to provide effective care, and focusing less on emotional discussions, considering their reluctance to open up and express themselves emotionally. In addition, we connected participants with community organizations that offer linguistically appropriate services, allowing them to receive more detailed guidance from experts such as elder law attorneys and social service agency staff fluent in their languages. These efforts were intended to reduce financial and language barriers and improve access to essential services.

We also respected participants’ cultural values related to health and caregiving. While providing accurate information about dementia and its care, we honored participants’ cultural beliefs, acknowledging and respecting the viewpoint that dementia and memory loss are natural aspects of aging. Additionally, we respected traditional family structures and communication patterns, acknowledging that open discussions about emotional issues might not always be the preferred approach.

Along with the written materials mentioned in the *Content* section, we used euphemized language during conversations to align with East Asian cultural beliefs. Specifically, we used terms such as “memory loss” instead of “dementia,” “discomfort and unhappiness” instead of “mental health,” “being uncomfortable” instead of “stigma,” and “gaining a few more pounds than average” instead of “obesity.” Finally, we prepared culturally related refreshments during visits to express respect and build interpersonal relationships. This small gesture not only helped foster trust and rapport with participants but also contributed to participant retention by making them feel more comfortable and understood by the intervention staff.

## Discussion

### Principal Findings

We identified cultural adaptation strategies to an evidence-based program as classified by the 5 adaptation elements of Day et al [2] and the timing and types of responses to adaptation from the FRAME [42]. Our description underscores the importance of cultural adaptation in dementia caregiver interventions, demonstrating how and when modifications in content, context, fidelity, engagement, and cultural competence are applied to enhance accessibility and relevance for Chinese and Korean American communities.

Unlike prior culturally adapted interventions, our approach is pivotal because we systematically adjusted and implemented counseling-based interventions, an approach that is relatively unfamiliar among Asian Americans, to fit the cultural and linguistic context of Korean and Chinese American groups. Asian Americans showed lower rates of mental health service utilization, driven by language barriers, stigma toward mental illness, limited culturally sensitive therapeutic options, and relationships between clients and health professionals [50]. Documentation of this adaptation process can be extended to cultural adaptation strategies applicable to community organizations and health care professionals in the mental health field, potentially contributing to reducing the mental health access gap in Asian communities.

Moreover, the NYUCI-ES applies a structured, theory-driven approach that integrates both the cultural adaptation framework [2] and parts of FRAME [42]. The framework of Day et al [2] highlights the multifaceted content-related changes involved in culturally adapting psychosocial interventions, whereas the FRAME brings attention to how and when the intervention adaptations occur across the implementation timeline. The integrated use of these frameworks allows for a systematic documentation of cultural adaptations throughout the implementation process. It can further provide an in-depth understanding of the cultural adaptation process in the NYUCI-ES, which was based not only on participants’ individual and environmental factors but also on cultural considerations.

Moreover, the NYUCI-ES embeds deeper cultural constructs, such as familism, filial piety, and stigma, into the program’s design, counseling process, and engagement strategies. Beyond preimplementation adjustments, NYUCI-ES uniquely documents ongoing, real-time adaptations informed by participant and community feedback. We also added culturally unique communication platforms for peer support and recognized “fictive kin” in family sessions. This work can move the field beyond stating that cultural tailoring matters to showing how it can be systematically and sustainably achieved.

### Lessons Learned From the Cultural Adaptation Process

Content and context modifications used in our adaptation were similar to those used in other culturally adapted interventions targeting East Asian cultures [9,37-41], including language translation and considerations of participants’ cultural values in program components and format. Approximately half of the Korean and Chinese American population whose origins are outside of the United States revealed limited English proficiency [51,52]. Considering the language barrier, providing information translated into the participant’s familiar language is essential to providing adequate support based on their needs [53]. This strategy not only creates comfort and familiarity with the intervention but also builds a foundation for trust and engagement. To provide effective support that can have a positive impact on the physical and emotional health of caregivers, it is essential to take into account the recipients’ cultural identity.

However, our adaptation process went beyond mere language translation. For instance, participants’ hesitancy toward involving family members in counseling was due to cultural beliefs about caregiving as a personal duty rather than a responsibility to be shared by their families or external resources [10]. To mitigate their reluctance, we reframed culturally sensitive wording, emphasized family harmony, and provided primary caregivers with practical support. Additionally, allowing participants to invite their fictive kin with similar cultural and situational backgrounds to the family counseling sessions could broaden their informal support network. Partnerships with community-based organizations also provided valuable insights into recruitment and retention, effective communication with the target population, and the exchange of study information and community resources, enabling us to offer participants culturally relevant resources and support.

Maintaining fidelity to the original intervention components while incorporating cultural adaptations was a key theme of our study. By involving the original intervention developer and clinical supervisor with experts in East Asian culture throughout the whole process, we ensured that core elements were preserved while making necessary cultural adjustments before and during the implementation. This balance is critical for maintaining the intervention’s evidence-based foundation while enhancing its cultural relevance.

Moreover, engagement strategies took into account the culturally grounded hesitancy in seeking help or sharing dementia care experiences with others [54]. A step-by-step approach with prolonged conversations with participants and financial compensation for completing assessments mitigated barriers to participation and fostered trust between participants and the intervention team.

Considering culturally rooted beliefs and norms is also important to sustain participants’ engagement. Efforts to enhance cultural competence included adjusting counseling approaches to Eastern caregiving norms and family dynamics. By prioritizing family harmony and using culturally appropriate language, we could discuss sensitive topics in a way that resonated with participants’ values and beliefs. Culturally adapted strategies are essential for recruiting and retaining individuals from diverse cultures to participate in research. The flexibility of the original NYUCI allowed us to provide person-centered, culturally tailored resources in participants’ preferred language during counseling sessions. As the topics of the counseling sessions were based on participants’ concerns or needs, it permitted ample flexibility to offer personalized support. This approach also fostered a stronger connection with the social worker providing counseling, which may have enhanced participant engagement and is anticipated to contribute to the overall effectiveness of the intervention.

In terms of the timing and types of response to adaptation, we used cultural adaptation strategies both proactively and reactively, before and during the study implementation. However, more reactive strategies were often conducted to increase participant engagement and to address participants’ cultural beliefs and viewpoints on dementia care and intervention (ie, cultural competence). Whereas cultural adaptation approaches are prepared proactively to reflect the targeted cultural content and context, ongoing reactive adjustments during the implementation period are necessary to address unexpected challenges promptly in real-world settings [42]. At the same time, these adaptations should remain the core value and components of the original intervention [42]. Day et al [2] also emphasized the pivotal role of fidelity in culturally adapting interventions and the need for consideration of fidelity at every stage of the adaptation process. Hence, there is a need to facilitate activities that can strengthen fidelity not only through observation and self-reports but also through a fidelity checklist and the examination of how modifications influence the intervention outcomes [42].

Another key lesson from our study was the limited desire for psychological support among participants. For example, caregivers in our study tended to prioritize practical caregiving strategies, such as learning symptom management and resource navigation, rather than emotional or mental health support for themselves. Additionally, some caregivers believed that counseling was solely for individuals with psychological disorders. In East Asian cultures, mental health issues are often stigmatized, sometimes perceived as a sign of personal failure or weakness [55], and dementia is often misunderstood as a mental illness. This stigma, combined with limited family support systems and language barriers, made it challenging for caregivers to access resources [9,56]. By emphasizing practical caregiving strategies during counseling and gradually incorporating emotional support, we could help caregivers feel more comfortable seeking help. Reframing counseling as a resource for both caregiving skills and caregiver well-being and providing culturally competent community resources aligning with their language and beliefs may further reduce stigma through caregivers’ willingness to accept concrete assistance. Furthermore, evaluating attitudes toward dementia throughout a study such as this could also help identify the effectiveness of our culturally adapted intervention in mitigating stigma and lowering barriers to intervention engagement.

Hesitancy to share personal experiences, which was a similar challenge addressed by Jang et al, could result from privacy concerns, fear of judgment, and limited familiarity with counseling among the East Asian population [55,57]. To address this, we encouraged participants to share their expectations and provide feedback during the counseling sessions, maintained regular contact with caregivers in the study via social media apps, built rapport through multiple interactions and clear explanations of staff roles, and kept encouraging caregivers to share their concerns and questions. By structuring discussions based on participants’ concerns and actively facilitating conversations, rather than expecting caregivers to initiate discussions, our approach could improve engagement and make support more accessible for caregivers from Eastern Asian cultural backgrounds.

### Strengths and Limitations

One of the distinctions of our adaptation strategy compared to other cultural adaptation studies is the flexible program delivery method. Considering the advancement of technology, using digital tools in dementia care has become inevitable [58]. In our adaptation of the NYUCI, we offered counseling sessions in person and through video or audio calls and modified the original intervention by integrating social media group support. These modifications are intended to help participants easily access culturally tailored resources and provide continuous peer support. Another strength is the active involvement of the original NYUCI developer and senior clinician in all phases to ensure maximum fidelity to the original while respecting and considering the cultural values of the target populations. This active engagement and supervision, both before and during the implementation, helped us make necessary cultural adaptations while preserving the original evidence-based intervention’s principles. In addition, instead of focusing solely on language translation or surface-level modifications, our approach combined comprehensive and theory-based adaptations. By considering multiple cultural factors while maintaining the original NYUCI’s structure, our intervention not only aligned with participants’ needs but also maintained the integrity of the original program. Moreover, the adaptation process went beyond individual decisions and was conducted collectively through group discussions. This collaborative process highlights the interactive decision-making process while paying close attention to the experience and response from social workers and other study personnel who directly contact participants.

There are several limitations to our observations. One key issue was the overlap among the concepts of the 5 key elements, which made it difficult to delineate specific adaptation strategies. For example, hiring personnel with the same background and training them in the culturally tailored intervention approach is one of the “context” modifications. This suggests a need for further refinement of cultural adaptation frameworks to improve their practical application in intervention design and implementation. Second, this analysis relied mostly on internal team meetings and self-reports rather than direct participant perspectives through the interview recordings, which may limit the diversity of viewpoints represented. Owing to the fact that the study is not yet complete, the effectiveness of these adaptations on implementation and outcomes has not yet been assessed. Although the achievement of our recruitment goals [11] may be indirect evidence of our success in the cultural adaptation approach, it is warranted to evaluate the strategies for cultural adaptation in future research. In addition, this study’s focus on specific subgroups of Asian caregivers in the New York City metropolitan area limits its generalizability to other subgroups of Asian Americans. There is a clear need for further research on other Asian subgroups and diverse geographical locations. Finally, as this is an ongoing study, we could not yet assess the effectiveness of the culturally adapted intervention. However, our approach already provides information to inform future development of culturally tailored dementia caregiver support programs. Assessment and refinement of these strategies will continue as we move forward.

### Implications for Future Culturally Adapted Interventions

A lack of culturally and linguistically competent resources can exacerbate systemic health care barriers among Asian American communities [59], indicating the need for greater cultural inclusivity [10]. Strategies to address this gap include further development and refinement of guidelines for implementing culturally sensitive support based on evidence-based interventions, training professionals in cultural competency, and integrating community-based resources in all aspects of the endeavor [53]. Flexible delivery methods, including technology use, can also support individual needs across generations and geographic distances. Currently, there are limited guidelines and frameworks for culturally adapted interventions in dementia caregiving [2]. Integrating theoretical frameworks while adapting interventions based on a specific culture is necessary to ensure cultural relevance and program fidelity. Finally, continued evaluation and refinement of cultural adaptation strategies are important. Our ongoing RCT of the NYUCI-ES targets a large sample and provides year-long support for each participant. The description of adaptation strategies in this study will contribute to the growing literature on the long-term effects of culturally adapted, evidence-based interventions.

### Conclusions

This study explored cultural adaptation strategies within an ongoing dementia caregiver intervention, NYUCI-ES, tailored to Chinese and Korean cultures. This approach can improve cultural sensitivity while preserving key components of an evidence-based intervention. Our future research will focus on evaluating the effectiveness of NYUCI-ES and the influence of adaptation approaches on participant outcomes and feedback. We anticipate that these findings will help guide the development of more inclusive and responsive caregiver support programs in dementia care.

## Abbreviations

FRAME: Framework for Reporting Adaptations and Modifications–Enhanced
NYUCI: New York University Caregiver Intervention
NYUCI-ES: New York University Caregiver Intervention–Enhanced Support
RCT: randomized controlled trial
TIDieR: Template for Intervention Description and Replication
